# Kinetic Effects of H_2_O_2_ Speciation on the Overall Peroxide Consumption at UO_2_–Water Interfaces

**DOI:** 10.1021/acsomega.2c01048

**Published:** 2022-04-27

**Authors:** Daniel Olsson, Junyi Li, Mats Jonsson

**Affiliations:** Department of Chemistry, KTH Royal Institute of Technology, Teknikringen 30, Stockholm SE-100 44, Sweden

## Abstract

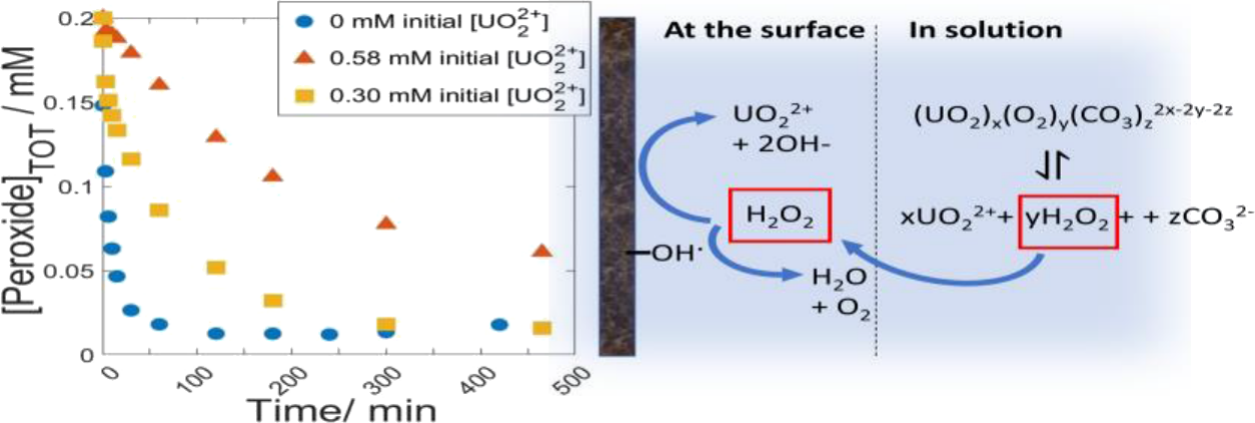

The interfacial radiation chemistry of UO_2_ is of key importance in the development of models to predict the corrosion rate of spent nuclear fuel in contact with groundwater. Here, the oxidative dissolution of UO_2_ induced by radiolytically produced H_2_O_2_ is of particular importance. The difficulty of fitting experimental data to simple first-order kinetics suggests that additional factors need to be considered when describing the surface reaction between H_2_O_2_ and UO_2_. It has been known for some time that UO_2_^2+^ forms stable uranyl peroxo-carbonato complexes in water containing H_2_O_2_ and HCO_3_^–^/CO_3_^2–^, yet this concept has largely been overlooked in studies where the oxidative dissolution of UO_2_ is considered. In this work, we show that uranyl peroxo-carbonato complexes display little to no reactivity toward the solid UO_2_ surface in 10 mM bicarbonate solution (pH 8–10). The rate of peroxide consumption and UO_2_^2+^ dissolution will thus depend on the UO_2_^2+^ concentration and becomes limited by the free H_2_O_2_ fraction. The rate of peroxide consumption and the subsequent UO_2_^2+^ dissolution can be accurately predicted based on the first-order kinetics with respect to free H_2_O_2_, taking the initial H_2_O_2_ surface coverage into account.

## 1 Introduction

Uranium dioxide (UO_2_) is the most common fuel material used in commercial nuclear reactors. While the unirradiated nuclear fuel has a low radioactivity, the formation of a small percentage of fission products and heavier actinides in the nuclear reactor leads to dramatically increased radioactivity that persists long after the fuel has been removed from the reactor.^1^ Disposal of the spent nuclear fuel is one of the major challenges in nuclear technology. A solution that has been widely accepted is permanent storage in deep geological repositories.^2,3^ In Sweden and Finland, the so-called KBS-3 concept will be applied, where the spent nuclear fuel is sealed in copper-coated cast iron canisters placed in the crystalline bedrock at a depth of around 500 m below the ground.^4^ The canisters will be embedded in compacted bentonite clay. In the event of multiple barrier failure, the fuel would come in contact with groundwater. UO_2_ has very low solubility in water but when oxidized into UO_2_^2+^, the solubility is enhanced by several orders of magnitude.^5,6^ Complexation with carbonate (typically present in groundwater in concentrations 1–10 mM, depending on the geographical site of the repository)^7,8^ increases the solubility further, favoring oxidative dissolution of the spent nuclear fuel matrix.^9^ In general, the groundwater conditions at the depth of the repository are expected to be more reducing than oxidizing.^6^ However, the intrinsic radioactivity of the spent nuclear fuel will induce groundwater radiolysis, resulting in the formation of reactive oxidants (HO^•^, HO_2_^•^, and H_2_O_2_) and reductants (e_aq_^–^, H^•^, and H_2_).^10−12^ For kinetic reasons, the oxidants will dominate the surface reactions. It has been demonstrated that the radiolytic oxidant to which oxidative dissolution of UO_2_-based fuel under repository conditions can mainly be attributed is H_2_O_2_.^13^ Hydrogen peroxide has been shown to react with a UO_2_ surface through the oxidation of U(IV) to U(VI) and catalytic decomposition on the oxide surface, leading to the formation of oxygen and water. The mechanisms can be described as follows:^14^
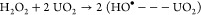
1
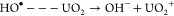
2
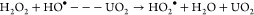
3
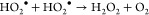
4

Both reactions have the surface-bound hydroxyl radical as a common intermediate. In water containing [HCO_3_^–^] > 1 mM, the oxidative dissolution of UO_2_ is limited by the one-electron oxidation described by reaction 2. For oxidative dissolution to occur, U(V) is further oxidized to U(VI). This could occur through a reaction with H_2_O_2_ or by disproportionation of two U(V) formed close to each other.

In systems with a high UO_2_ surface area to solution volume ratio (SA/V), the kinetics of H_2_O_2_ consumption is expected to be first order with respect to H_2_O_2_ as the surface coverage is expected to become negligible. However, first-order fitting of experimental data has often resulted in low accuracy;^15,16^ hence, the rate of consumption cannot be explained by strict first-order kinetics. The rate of H_2_O_2_ consumption at a given (measured) H_2_O_2_ concentration has been shown to be dependent on the initial H_2_O_2_ concentration, in sharp contrast to what is expected for first-order kinetics. This observation has previously been attributed to an irreversible alteration of the UO_2_ surface, where it was shown that the reactivity of UO_2_ pellets changed slightly over consecutive exposures.^17^ However, there are other possible explanations that are yet to be explored.

It is well known that UO_2_^2+^ forms stable uranyl-peroxo-carbonato complexes in solutions containing bicarbonate and hydrogen peroxide.^9,18−20^ Several species have been identified, and the speciation of the system will depend on the concentrations of the solutes, ionic strength, and pH. In general, the equilibrium between a complex and uncomplexed peroxide can be described as follows:^20^
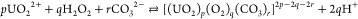
5

Acid–base equilibria must also be accounted for to fully describe proton exchanges related to reaction (5). The pKa values for (HCO_3_^–^/CO_3_^2–^) and (H_2_O_2_/HO_2_^–^) are 10.34 and 11.75, respectively.^21,22^

Despite the fact that the existence of uranyl-peroxo-carbonato complexes has been known for quite some time, they have not been accounted for when discussing the kinetics and the mechanism of the reaction between H_2_O_2_ and UO_2_. Instead, the peroxide concentrations measured in such systems are referred to as [H_2_O_2_],^15^ that is, free hydrogen peroxide. The equilibrium constants for the dominant complexes have been reported,^20,23^ allowing for the simulation of the equilibrium concentrations of peroxide species based on thermodynamic stability. The uranyl-peroxo-carbonato complexes are negatively charged and are therefore expected to have a lower affinity toward the negatively charged UO_2_ surface under alkaline conditions. Information regarding the reactivity of the peroxo-ligands in uranyl peroxo-carbonato complexes is limited. One of the few exceptions is a study by Chung et al.,^24^ reporting decomposition rate constants for the complex UO_2_(O_2_)(CO_3_)_2_^4–^ on various metal oxides.

In this work, we have experimentally explored the impact of H_2_O_2_-speciation on the kinetics and mechanism of H_2_O_2_-induced oxidative dissolution of UO_2_ in aqueous systems by varying the initial UO_2_^2+^ concentration. In addition, the impact of H_2_O_2_-speciation on the kinetics and mechanism of catalytic decomposition of H_2_O_2_ on ZrO_2_ was studied in the same way. Speciation calculations have been employed in order to estimate the relative fractions of peroxide species at different stages of the reactions.

## 2 Materials and Methods

### 2.1 Caution

Although the radioactivity of natural uranium (prior to its use in a nuclear reactor) is low, safety precautions regarding work with radioactive materials should be followed. Experiments involving uranium should only be conducted by trained staff and take place in facilities appropriate for the handling and storage of radioactive materials.

### 2.2 Exposures

The chemicals used throughout the experiments were of reagent grade or higher. All exposures were carried out in cylindrical glass vessels under N_2_ purging, using 50 mg of UO_2_ powder (supplied by Westinghouse Electric Sweden AB) in 30 mL of 10 mM bicarbonate solution (18.2 MΩ cm, Merck MilliQ). Sample volumes of 1 mL (or 2.5 if formaldehyde was analyzed) were removed from the reaction vessels at each point of measurements. The samples were filtered through 0.2 μm cellulose acetate syringe filters prior to analysis. The specific surface area of the powder had previously been determined as 4.6 ± 0.2 m^2^ g^–1^, and the oxidation state was determined to be hyper stoichiometric UO_2.3_.^15^ Before exposure, the UO_2.3_ powder was washed in 10 mM bicarbonate solution to remove preoxidized U(VI) from the surface. The washing process was carried out in five repetitions under N_2_ purging, during which the solution was magnetically stirred for 5 min and replaced after sedimentation of UO_2.3_, as indicated by a visibly clear solution. Before the replacement of the final washing solution with the solution used in the experiment, the uranyl concentration was measured to confirm uranyl from preoxidized U(VI) remained under the detection limit (<1 μM). The UO_2_^2+^/H_2_O_2_/HCO_3_^–^(CO_3_^2–^)-solutions were prepared by dissolving various amounts of UO_2_(NO_3_)_2_ × 6H_2_O in washed UO_2.3_ powder solutions (10 mM bicarbonate). Exposures were started as H_2_O_2_ was introduced to the systems.

Differences in the peroxide speciation at various concentrations of UO_2_^2+^ were simulated using SPANA,^25^ with ionic strength correction based on the Specific Ion-Interaction Theory (SIT) model.^5^ The stability constants used in this work are the ones presented by Zanonato et al.^18^ In a recent work, simulations based on the same set of constants were shown to be in good agreement with experimental observations using ^13^C NMR under similar conditions (0.2 mM initial H_2_O_2_ in 10 mM HCO_3_^–^).^19^

### 2.3 Spectrophotometric Measurements with UV–vis

Concentrations of UO_2_^2+^, H_2_O_2_, and formaldehyde were measured with UV–vis absorption spectroscopy, using a Thermo Scientific Genesys 20 spectrophotometer. Uranyl concentrations were determined directly with the Arsenazo III method.^26,27^ The absorbance of the U(VI)-(1,8-dihydroxynaphthalene-3,6-disulphonic acid-2,7-bis[(azo-2)-phenylarsonic acid]) complex was measured at 653 nm. The reaction was carried out by mixing 60 μL 1 M HCl and 40 μL 16 wt % arsenazo-III reagent solution with 1.5 mL of diluted (100–200 μL) sample directly in the cuvette. H_2_O_2_ concentrations were determined indirectly with the Ghormley triiodide method,^28^ by mixing 1.8 mL of diluted (100–200 μL) sample with 100 μL of 1 M potassium iodide and 100 μL of 1 M acetate/acetic acid buffer containing molybdate as a catalyst. The absorbance of I_3_^–^ (formed in 1:1 ratio with reduced H_2_O_2_) was measured at 360 nm. It should be mentioned that the triiodide method was tested for solutions of various concentrations of UO_2_^2+^ (0–4 mM). The measured concentration corresponded to the total amount of added H_2_O_2_ regardless of speciation. This is expected as the complex species will be converted back to H_2_O_2_ when the sample volumes are diluted in purified water (18.2 MΩ cm, Merck MilliQ) prior to measurement. The concentration measured with the triiodide method will henceforth be referred to as [peroxide], as to not be confused with the fraction of free H_2_O_2_ present in the UO_2_ and ZrO_2_ powder solutions during exposures.

The formation of surface-bound hydroxyl radicals following the decomposition of H_2_O_2_ on ZrO_2_ was analyzed indirectly via the formation of formaldehyde (formed as one of the final products when tris(hydroxymethyl)aminomethane (Tris) is used as a radical scavenger). The produced formaldehyde was measured using a modified version of the Hantzsch reaction,^29^ where formaldehyde reacts with acetoacetanilide and ammonia. Because of a larger sample volume required for the Hantzsch reaction than the 1 mL otherwise used, a volume of 2.5 mL sample was removed from the glass vessel for each point in time where concentrations were measured in the ZrO_2_ powder solutions. The Hantzsch reaction was carried out in glass tubes by mixing 5 mL of 2 M ammonium acetate, 2 mL of 0.2 M acetoacetanilide, 2 mL of ethanol, and 1 mL of sample (filtered through 0.2 μm cellulose acetate filters). The reaction was carried out for 20 min in a heating bath at 313 K. The product, a dihydropyridine derivative, was measured at the absorbance maximum, occurring at 368 nm.^29^

## 3 Results and Discussion

### 3.1 Effect of Speciation on the Consumption of H_2_O_2_ and UO_2_^2+^ Dissolution

**Figure 1:**
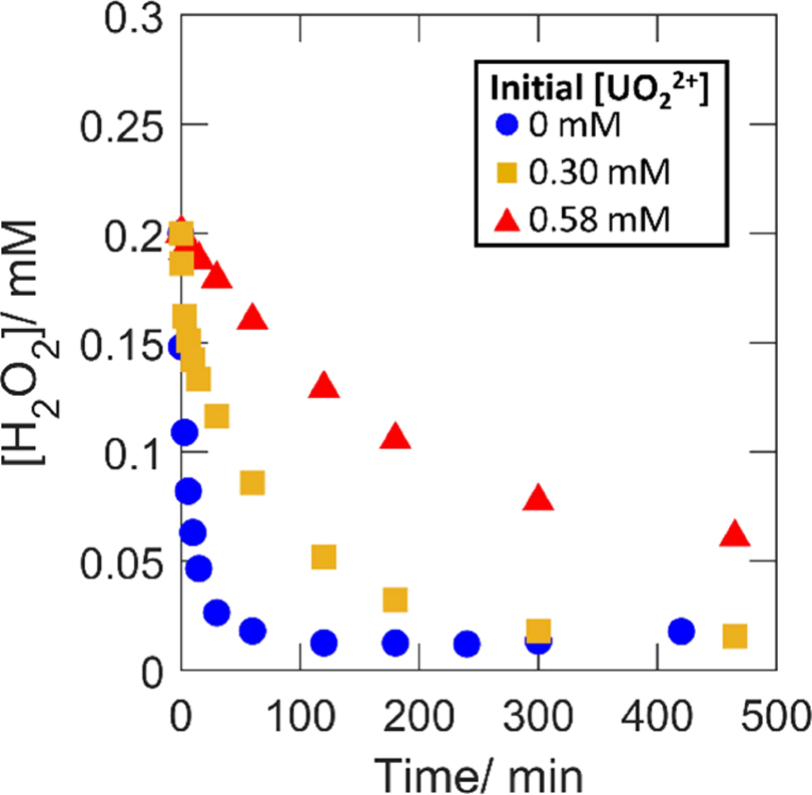
Peroxide concentrations as functions of exposure time for 50 mg of UO_2.3_ powder in 30 mL of 10 mM HCO_3_^–^ with 0.2 mM initial [H_2_O_2_] and varied initial [UO_2_^2+^].

**Figure 2:**
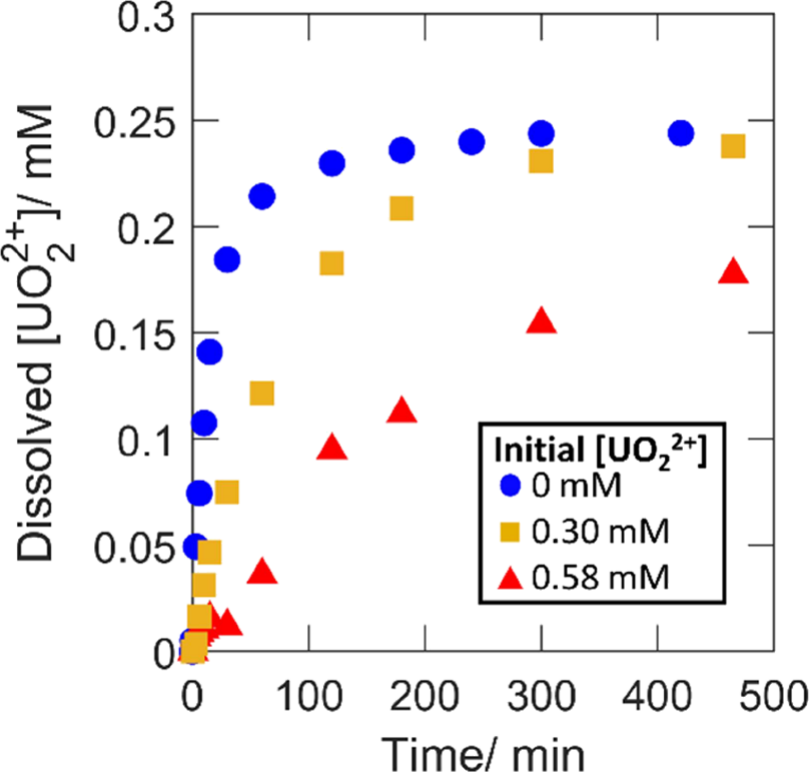
Dissolved UO_2_^2+^ as functions of exposure time for 50 mg of UO_2.3_ powder in 30 mL of 10 mM HCO_3_^–^ with 0.2 mM initial [H_2_O_2_] and varied initial [UO_2_^2+^].

**Figure 3:**
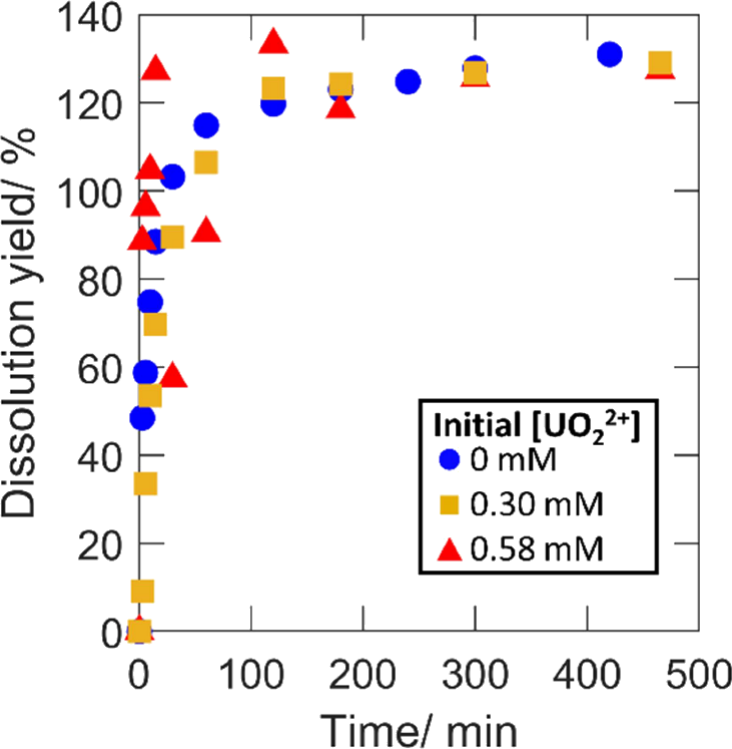
Cumulative dissolution yields as functions of exposure time for 50 mg of UO_2.3_ powder in 30 mL of 10 mM HCO_3_^–^ with 0.2 mM initial [H_2_O_2_] and varied initial [UO_2_^2+^].

In order to vary the initial H_2_O_2_ speciation, experiments were performed using initial UO_2_^2+^ concentrations of 0, 0.30, and 0.58 mM. These concentrations were selected based on thermodynamic calculations to correspond to initial free H_2_O_2_ fractions of 1, 0.5, and 0.1 based on the set conditions of 10 mM bicarbonate and the initial 0.2 mM H_2_O_2_. Total peroxide concentrations, dissolved UO_2_^2+^ concentrations (i.e., total concentration with the initial concentration subtracted), and dissolution yields as functions of reaction time are presented in Figures 1, 2, and 3, respectively. Dissolution yields exceeding 100% are expected based on the hyperstoichiometric state of the powder. For UO_2.3_, the dissolution yield would reach a maximum at ∼140% based on the already higher oxidation state of the uranium.

From Figure 1, it is clear that the rate of peroxide consumption is significantly reduced by the presence of uranyl in the solution. The rate decreases with increasing initial uranyl concentration. The same effect is observed for the rate of UO_2_^2+^ dissolution shown in Figure 2. From the comparison of the dissolution yields in Figure 3, it is quite clear that the uranyl-peroxo-carbonato complexes have little or no influence on the final amount of UO_2_^2+^ dissolved per amount of peroxide consumed, that is, the final dissolution yield is not affected by the initial presence of UO_2_^2+^. Fluctuations of the dissolution yield at low exposure times for 0.58 mM initial uranyl are expected as the result of uncertainties when measuring small changes to a relatively high total concentration. The yield becomes more certain as the dissolved amount increases. It should be noted that the dissolution yield depends on the total carbonate concentration. At total carbonate concentrations significantly below 10 mM, the dissolution yield will not reflect the competition between UO_2_ oxidation by H_2_O_2_ and surface catalyzed decomposition of H_2_O_2_ but also be affected by limitations in the solubility of oxidized UO_2_.

The observed suppression of the rates with added uranyl, along with the similar dissolution yields implies that the reaction mainly occurs between free H_2_O_2_ and the UO_2_ surface. At higher initial UO_2_^2+^ concentrations, the fraction of free H_2_O_2_ is lower, and therefore, the overall rates of peroxide consumption as well as UO_2_^2+^ release are lower. Given the mechanism for the reaction between H_2_O_2_ and UO_2_ (reactions 1−4), the presence of uranyl-peroxo-carbonato complexes would favor higher dissolution yields by decreasing the concentration of free H_2_O_2_. However, such a trend may not be possible to observe under these conditions because the dissolution yield is already at or near its maximum.

**Figure 4:**
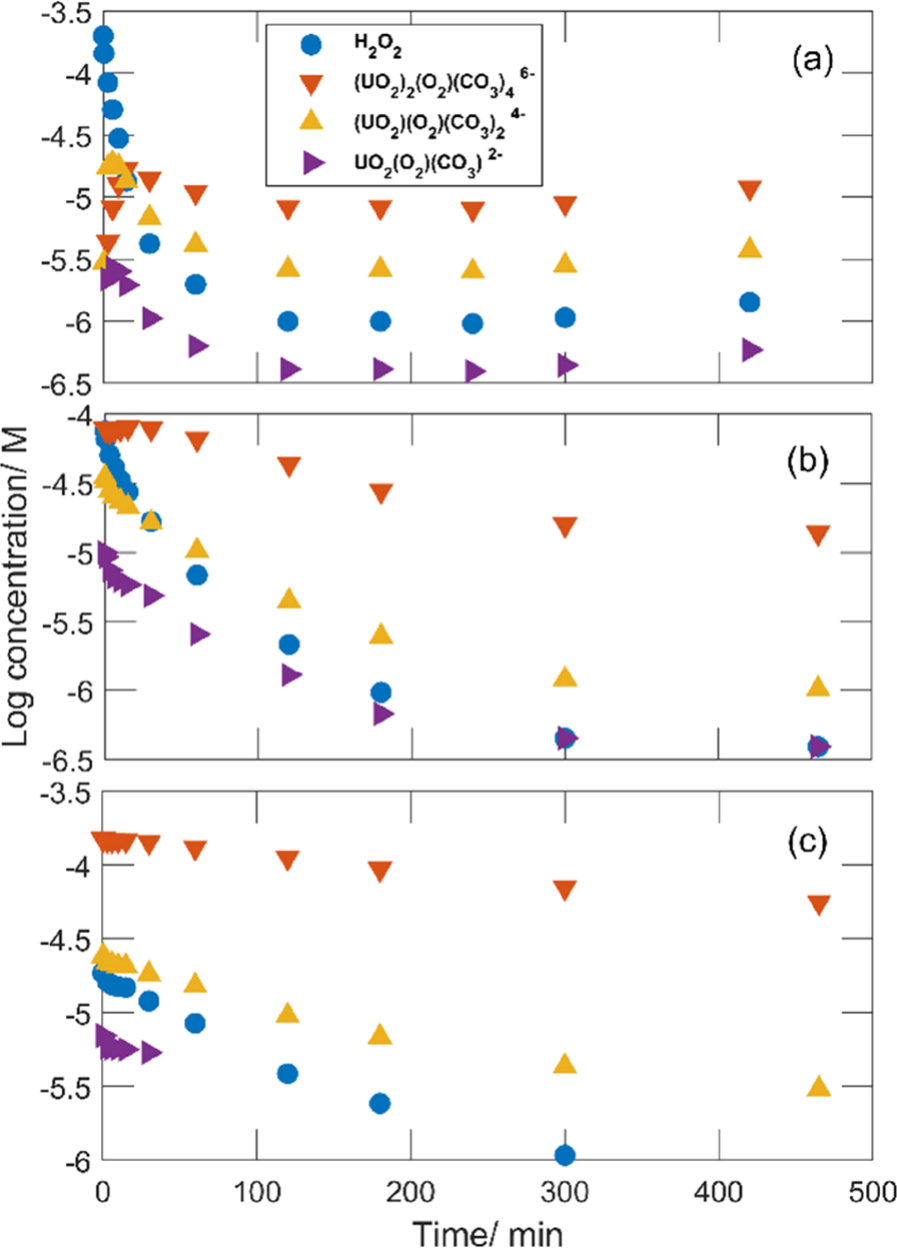
Simulations of the log equilibrium concentrations for the dominant peroxide species as functions of exposure time in 10 mM bicarbonate solutions containing 0.2 mM H_2_O_2_ and 0 mM (a), 0.3 mM (b), and 0.6 mM (c) UO_2_^2+^ at the start of the exposure. The calculations were performed using concentrations presented in Figures 1 and 2 and pH 9.2.

To analyze the speciation as a function of time in the three experiments, we simulated the thermodynamic equilibrium concentrations of the dominant peroxide species as functions of exposure times using SPANA. pH was measured at the start and at the end of each exposure, and the average (∼pH 9) was assumed when performing the simulations. The simulated concentrations for 0.2 mM initial H_2_O_2_ and 0, 0.30, and 0.58 mM initial [UO_2_^2+^] are presented in Figure 4a–c, respectively.

As can be seen in Figure 4 a, the dominating peroxide species in the case where no UO_2_^2+^ is added is initially free H_2_O_2_ while in the cases where the initial [UO_2_^2+^] exceeds 0.3 mM (Figure 4b,c), the peroxide is expected to predominantly exist in the form of (UO_2_)_2_(O_2_)(CO_3_)_4_^6–^. The fraction of free H_2_O_2_ decreasing with increasing initial UO_2_^2+^ is in qualitative agreement with the conclusion above that the reaction occurs between free H_2_O_2_ and UO_2_ as reflected by the relative rates.

### 3.2 Kinetic Analysis of Reference Experiments with Varied Initial [H_2_O_2_]

**Figure 5:**
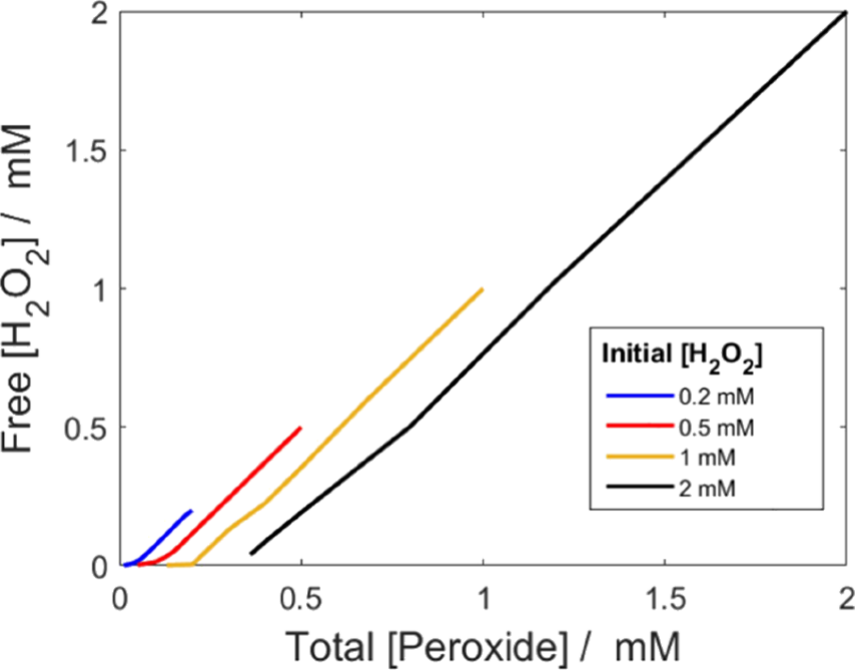
Calculated free H_2_O_2_ concentration as a function of the total peroxide concentration for UO_2.3_ powder in 10 mM HCO_3_^–^, with varied initial H_2_O_2_ concentrations. Calculations were based on the reported uranyl and peroxide concentrations (see reference, Figure 1)^15^ and an assumed pH 9.

To quantitatively explore the concept of nonreactive peroxo-complexes, we analyzed the kinetics of peroxide consumption for a set of previously published data.^15^ The data set includes H_2_O_2_ and UO_2_^2+^ concentrations as functions of reaction time for UO_2.3_ powder in 10 mM HCO_3_^–^ solutions, exposed to 0.2, 0.5, 1.0, and 2.0 mM initial H_2_O_2_ concentrations. It was found that the peroxide consumption rate at a given peroxide concentration varied significantly depending on the initial concentration of H_2_O_2_. The proposed explanation in the original work is an alteration of the reactive interface, leading to a change in reactivity.^15^ Here, we consider overestimations of free H_2_O_2_ available to the UO_2_ surface as a plausible explanation for this observation as the total peroxide measured was thought to exclusively be in the form of H_2_O_2_. A comparison of the calculated free H_2_O_2_ vs the total peroxide concentration is presented in Figure 5. The concentration of free H_2_O_2_ is based on speciation calculations using the reported UO_2_^2+^ and total peroxide concentrations (measured with the triiodide method).^15^

In Figure 5, it is clear that the H_2_O_2_ available to the surface at a given measured peroxide concentration is expected to vary based on the initial H_2_O_2_ (i.e., on the amount of dissolved UO_2_^2+^). It is interesting to note that the expected effect is a parallel shift of the curves in Figure 5, to which the consumption rate would correspond given first-order kinetics with respect to the fraction of free H_2_O_2_.

Although the parallel shift attributed to speciation could account for part of the experimental observation, it is obvious that it is not the only reason. Assuming the rate of H_2_O_2_ consumption to be directly proportional to the concentration of free H_2_O_2_, a plot of the rate as a function of total peroxide concentration would look like that given in Figure 5. However, in the original work, it is quite clear that the slopes of the individual curves (for each initial H_2_O_2_ concentration) differ from each other (the slopes decrease with increasing initial H_2_O_2_ concentration).

Differences in pH would be one possible reason for the difference in slopes. Simulations of the speciation for the systems containing 0.2 and 2 mM initial H_2_O_2_ at various pH suggest that the slopes of [H_2_O_2_] vs [peroxide] (presented in Figure 7) would decrease slightly with increasing pH within the pH range 7–11 (see Tables S1–S4 in the Supporting Information for comparison). In general, we have observed a slight increase in pH as the reaction progresses but not to the extent that would explain the observed differences in slopes.

**Figure 6:**
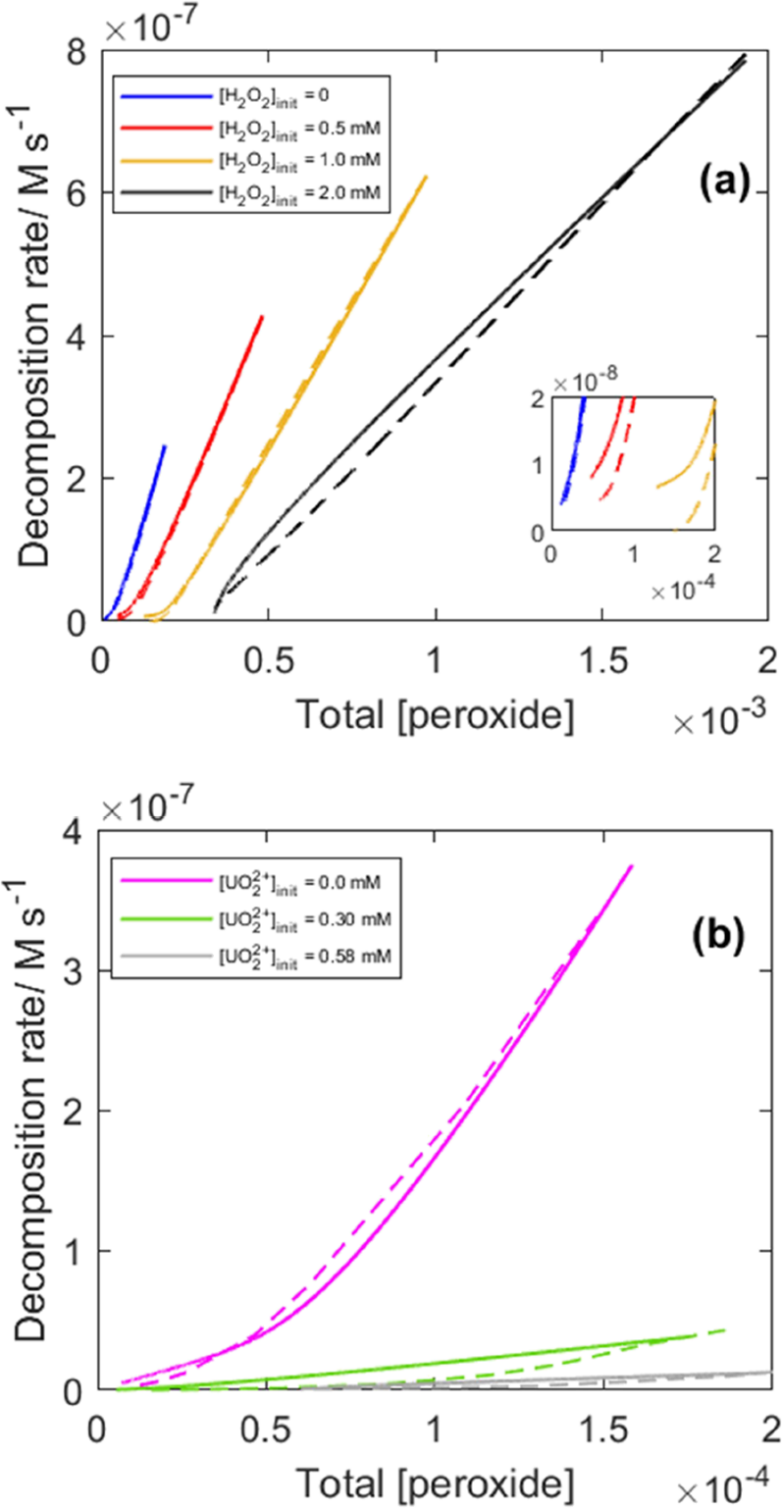
Comparisons of calculated rates based on first-order kinetics with respect to free [H_2_O_2_] (dashed lines) and the rates obtained by exponential fitting of the raw data for (a) a reference data set with varied initial [H_2_O_2_], SA/V = 5400 m^–1^ and (b) a set with varied initial [UO_2_^2+^], SA/V = 9000 m^–1^.

In the original work, it was demonstrated that the initial rate of H_2_O_2_ consumption depends on the initial H_2_O_2_ concentration through a relationship that can be given by a Freundlich adsorption isotherm. This suggested that the initial H_2_O_2_ consumption rate is proportional to the surface density of adsorbed H_2_O_2_. In order to model the kinetics of the system taking into account that the rate constant for the reaction between H_2_O_2_ and the surface displays some dependence on the initial H_2_O_2_ concentration, we determined the initial rate of H_2_O_2_ consumption from the derivative of the multiexponential function obtained from the fitting to experimental data. The first order rate constant to be used in the simulation was then derived by dividing the initial rate with the initial H_2_O_2_ concentration. The resulting rate constants are listed in the Supporting Information (see Table S5). By multiplying the free H_2_O_2_ concentrations derived from the speciation calculation (Figure 5) with the calculated rate constants, we obtain the expected rates of peroxide consumption. For comparison, both the calculated rates and the experimentally determined rates for the reference experiment where the initial H_2_O_2_ concentration was varied are plotted in Figure 6a.

A similar plot is shown in Figure 6b for the data set where the initial uranyl concentration was varied (data presented in Figures 1^2^,3). It should be noted that the SA/V is higher in these systems. Hence, the first-order rate constants derived for the first set of experiments cannot be used here. The higher SA/V also results in larger initial drops in peroxide concentration because of the fast adsorption of H_2_O_2_. This has a significant impact on the fitting of the peroxide concentration over exposure time. For this reason, the initial point has been excluded when fitting the data to improve the overall fit. Because two of the systems shown in Figure 6b has a significant amount of the peroxide in various uranyl-peroxo-carbonato complexes the first-order rate constants were calculated as the consumption rate divided by the calculated free [H_2_O_2_] after the initial rapid adsorption step.

As can be seen in Figure 6a, the calculated rates based on first-order kinetics with respect to the fraction of free H_2_O_2_ are in very good agreement with the experimental data when the pseudo first-order rate constants are calculated separately. This implies that both H_2_O_2_ speciation and the adsorption dependent pseudo first-order rate constant must be accounted for when describing the kinetics of H_2_O_2_ consumption on UO_2_.

Notably, there is less agreement between the experimental rates and the rates based on speciation calculations for the 2 mM initial H_2_O_2_ exposure compared to the exposures at lower initial H_2_O_2_ concentrations. In addition, the experimental rate has a dependence on the peroxide concentration different from that of the lower exposures as the rate appears to reach zero with a significant amount of peroxide remaining in solution. This could be a problem related to the fitting of the experimental data as the fit largely relies on the last point of the measured [peroxide] (see Figure S1d).

In general, the rates estimated on the basis of speciation calculations and first-order rate constants appear to be lower than the experimental rates in cases where the fraction of free H_2_O_2_ is low as can be seen toward the end of the exposure with lower initial H_2_O_2_ concentration (see the scale-up in Figure 6a). Underestimated rates are also obtained as the reaction progresses for the two cases where uranyl was added prior to exposure, as can be seen in Figure 6b (a scale-up is available as Supporting Information, see Figure S6). This could be attributed to uncertainties in the stability constants used for the speciation calculations. The calculated relative fractions are sensitive to uncertainties in the stability constants, as was demonstrated by Zanonato et al.^20^

### 3.3 Effects of Uranyl Peroxo-Carbonato Speciation on the Decomposition of H_2_O_2_ on ZrO_2_

**Figure 7:**
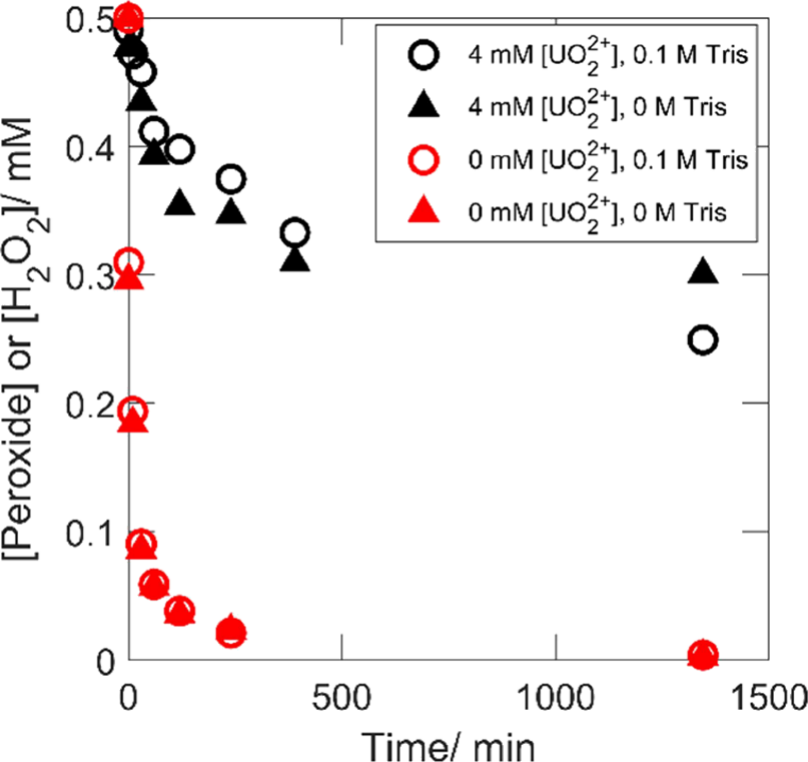
Total [peroxide] ([H_2_O_2_] in the absence of U (red keys)) as functions of exposure time for ZrO_2_ powder in 0.1 M HCO_3_^–^ with 0.5 mM H_2_O_2_, 0.1 M/ 0 M Tris and 4 mM/0 mM [UO_2_^2+^].

H_2_O_2_ reacts with ZrO_2_ by catalytic decomposition only. The ZrO_2_ system is therefore very suitable for studying the effect of H_2_O_2_ speciation on surface-catalyzed decomposition of H_2_O_2_. It has been shown in several relatively recent studies^30−33^ that the surface-bound hydroxyl radical is formed also in the catalytic decomposition of H_2_O_2_ on ZrO_2_. This has been demonstrated by using various radical scavengers. One of them is tris(hydroxymethyl)aminomethane (Tris), which produces formaldehyde upon reaction with the hydroxyl radical.^34^ Formaldehyde can readily be detected and thereby probe the accumulated hydroxyl radical production. Two sets of experiments were performed. In the first set, the H_2_O_2_/HCO_3_^–^(CO_3_^2–^)/ZrO_2_ system was investigated with and without Tris, and in the second set, the same systems with initially added UO_2_^2+^ were investigated. The peroxide concentrations ([H_2_O_2_] in the absence of UO_2_^2+^) as functions of exposure time are presented in Figure 7.

**Figure 8:**
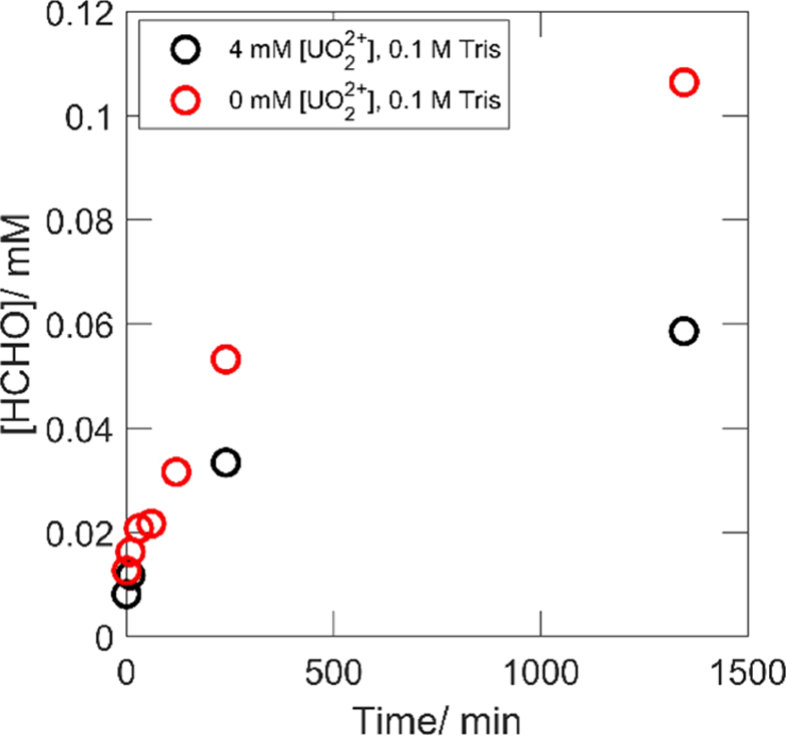
Detected formaldehyde as functions of exposure time in the presence and absence of 4 mM UO_2_^2+^ with 0.1 M Tris as an OH-radical scavenger.

As can be seen in Figure 7, H_2_O_2_ is rapidly consumed in the absence of UO_2_^2+^, and the presence of Tris does not appear to influence the rate at which H_2_O_2_ is consumed on ZrO_2_. The presence of UO_2_^2+^ clearly has a significant suppressing effect on the kinetics of peroxide consumption on ZrO_2_ where catalytic decomposition is the only reaction path. After 22 h and 26 min when the last measurement was made, approximately half of the peroxide had been consumed. The detected formaldehyde in the presence and absence of UO_2_^2+^ as functions of exposure time is presented in Figure 8.

The detection of formaldehyde in Figure 8 indicates the formation of surface-bound hydroxyl radicals in the presence and absence of UO_2_^2+^ that continues to be scavenged by Tris also after most of the H_2_O_2_ has been consumed. Interestingly, the final formaldehyde yields appear similar for the two sets because roughly twice as much formaldehyde was detected in the absence of UO_2_^2+^, where twice the amount of H_2_O_2_ was converted. Hence, the reaction mechanism would appear to be the same involving the formation of surface-bound hydroxyl radicals. Again, this would imply that the reaction proceeds via the fraction of free H_2_O_2_ and that the complexes merely act as a sink for free H_2_O_2_.

## 4 Conclusions

The formation of uranyl peroxo-carbonato complexes suppresses the rate of peroxide consumption by acting as temporary sinks for H_2_O_2_ while surface reactions on UO_2_ and ZrO_2_ likely proceed via the fraction of free H_2_O_2_. The peroxide in the various complex forms present in 10 mM bicarbonate show little or no reactivity toward the UO_2_ and ZrO_2_ surfaces under alkaline conditions (pH 8–10), as supported by the similar dissolution yields and the ratio between scavenged OH-radicals and the amount of consumed peroxide, regardless of the speciation. The very low reactivity of the complexes can largely be attributed to the electrostatic repulsion between the negatively charged complexes and the negatively charged surface. The kinetics of H_2_O_2_ consumption on UO_2_ surfaces in HCO_3_^–^ containing aqueous systems can be correctly reproduced using the fraction of free H_2_O_2_ determined from speciation calculations and the pseudo first-order rate constant given by the Freundlich isotherm for H_2_O_2_ on UO_2_.

## Supporting Information Available

Fittings of [peroxide] vs time data to multi-exponential equations (Figures S1 and S2), consumption rates vs time (Figure S3), consumption rates vs [peroxide] (Figure S4), measured uranyl concentrations vs exposure times related to the dissolution presented in Figure 2 (Figure S5), scale-up of Figure 6b (Figure S6), calibration data for the Arsenazo-III method (Figure S7), speciation calculation data for pH 7, 8, 9, 10, and 11 (Tables S1–S4), and pseudo first-order rate constants used for calculations of the rates shown in Figure 8 (listed in Table S5) (PDF)
