# Supplementary material for: Kinetic Effects of H2O2 Speciation on the Overall Peroxide Consumption at UO2–Water Interfaces
Source: ACS Omega. 2022 Apr 27;7(18):15929–35. doi: 10.1021/acsomega.2c01048 (PMC9097187; doi:10.1021/acsomega.2c01048)
Supplement: Supplementary file 1 — ao2c01048_si_001.pdf [file ao2c01048_si_001.pdf]

Supporting information for article ”Kinetic Effects of H_2O_2 Speciation on the Overall Peroxide Consumption at UO_2-water interfaces”

Daniel Olsson*[a], Junyi Li[a] and Mats Jonsson[a]

[a]Department of Chemistry, School of Engineering Sciences in Chemistry, Biotechnology and Health, KTH Royal Institute of Technology, SE – 100 44 Stockholm, Sweden

*Corresponding author.
E-mail: daniols@kth.se (D. Olsson)

Fitting of [peroxide] vs time

Experimental data of [Peroxide] as a function of time, fitted to an exponential expression of the form [peroxide] = $a \times e^{(b \times t)} + c \times e^{(d \times t)}$. The constants a, b, c and d were numerically optimized using a Trust-region algorithm. Fitting of the data for reference experiments (Barreiro Fidlago *et. al*) in Figure S1, followed by corresponding fittings of experimental data

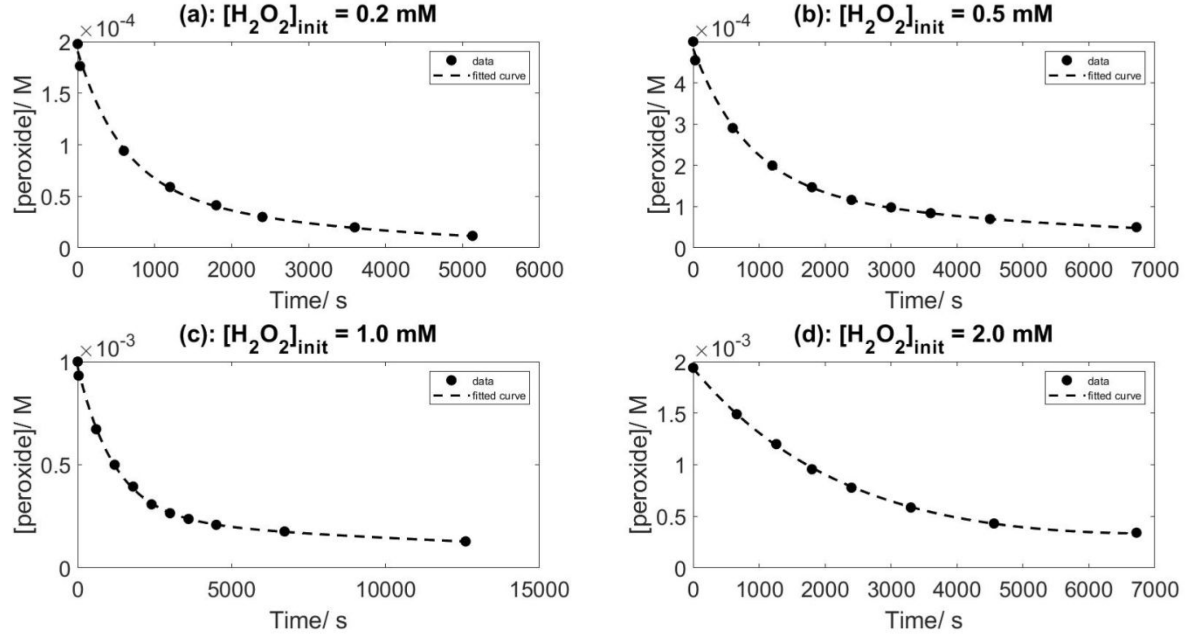

Figure S1. Multiexponential fitting for the systems containing 10 mM bicarbonate varied initial [H_2O_2], with SA/V = 5400 m^{-1} (100 mg $UO_{2.3}$ powder in 100 ml solution). Adapted with permission from [Alexandre Barreiro Fidalgo, Yuta Kumagai & Mats Jonsson (2018) The role of surface-bound hydroxyl radicals in the reaction between H_2O_2 and UO_2, Journal of Coordination Chemistry, 71:11-13, 1799-1807, DOI: 10.1080/00958972.2018.1466287]. Copyright [2018] [Alexandre Barreiro Fidalgo, Yuta Kumagai & Mats Jonsson]).

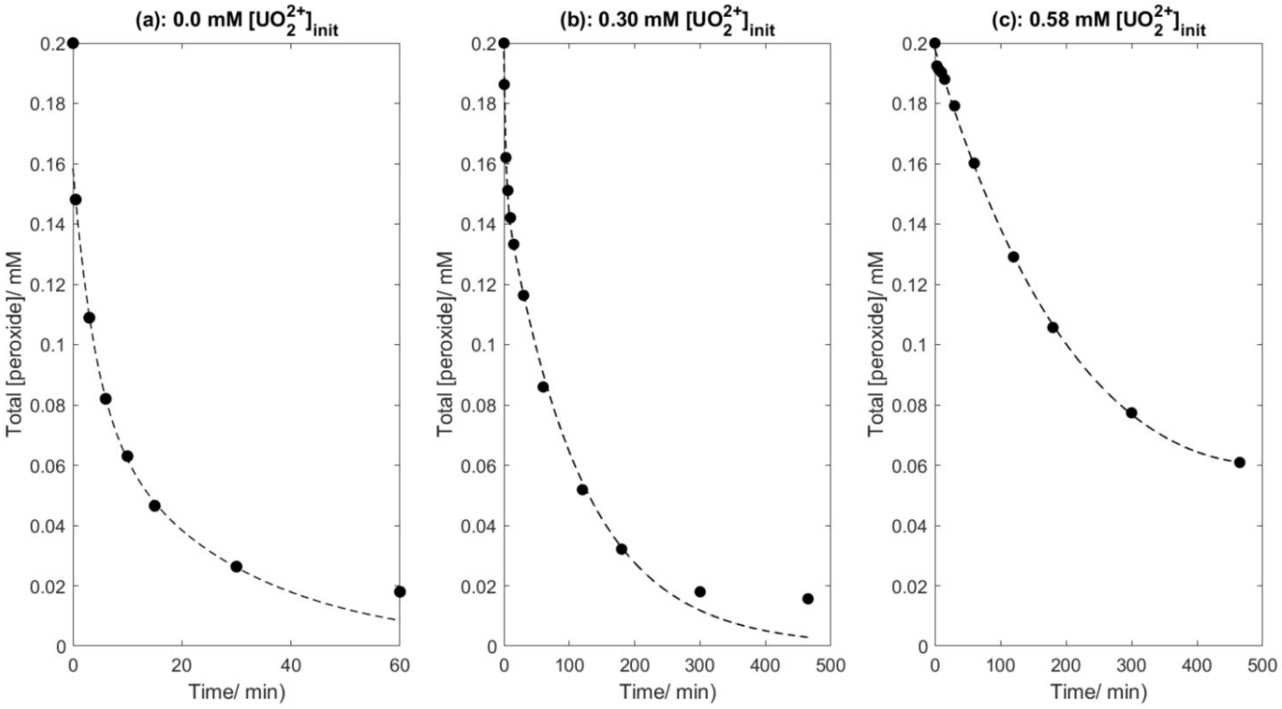

Figure S2. Multiexponential fitting of [peroxide] vs time for systems containing 10 mM bicarbonate, 0.2 mM initial [H_2O_2] and varied initial [UO_2^{2+}], with SA/V = 9000 m^{-1} (50 mg $UO_{2.3}$ powder in 30 ml solution).

The experimental rates corresponding to the derivatives of the exponential fittings presented in S1, are presented in Figure S3.

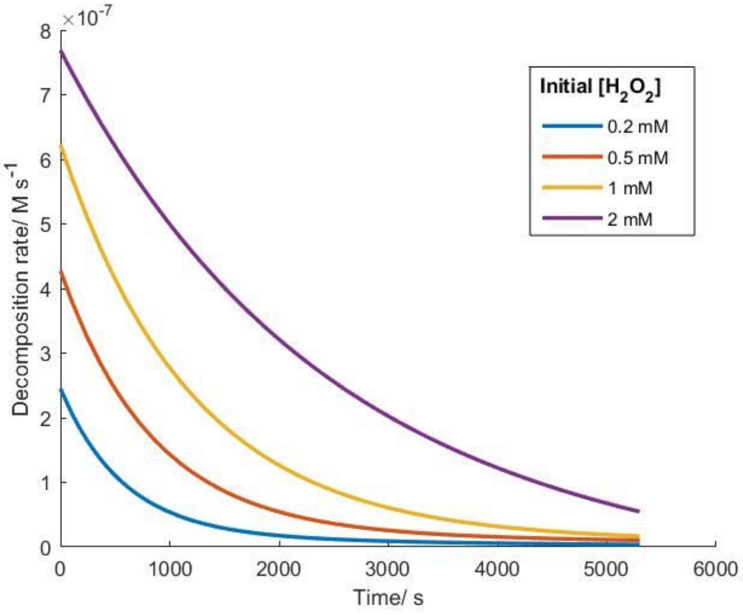

Figure S3. Consumption rates vs time from the derivatives of the [peroxide] vs time functions presented in S1.

The rates have been expressed as functions of [peroxide] by interpolated values obtained from the fitted functions (shown in Figure S1) and their derivatives (shown in Figure S2), using a step size of 1 second within the time interval of each experiment. The rates as functions of [peroxide] is presented in Figure S3.

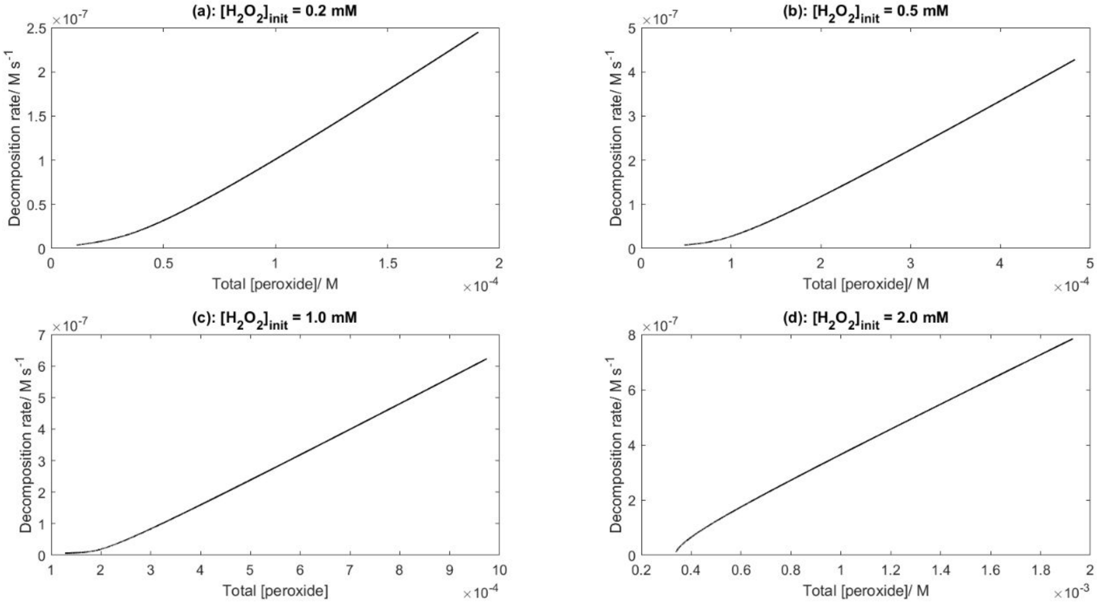

Figure S4. Interpolated rates as functions of interpolated [peroxide] with values obtained from the [peroxide] vs time function and the derivative (rate function) with the step size of 1 s between each point.

Measured uranyl concentrations corresponding to data presented in Figure 2

Measured uranyl concentrations (dissolved + added uranyl) are presented in Figure S4.

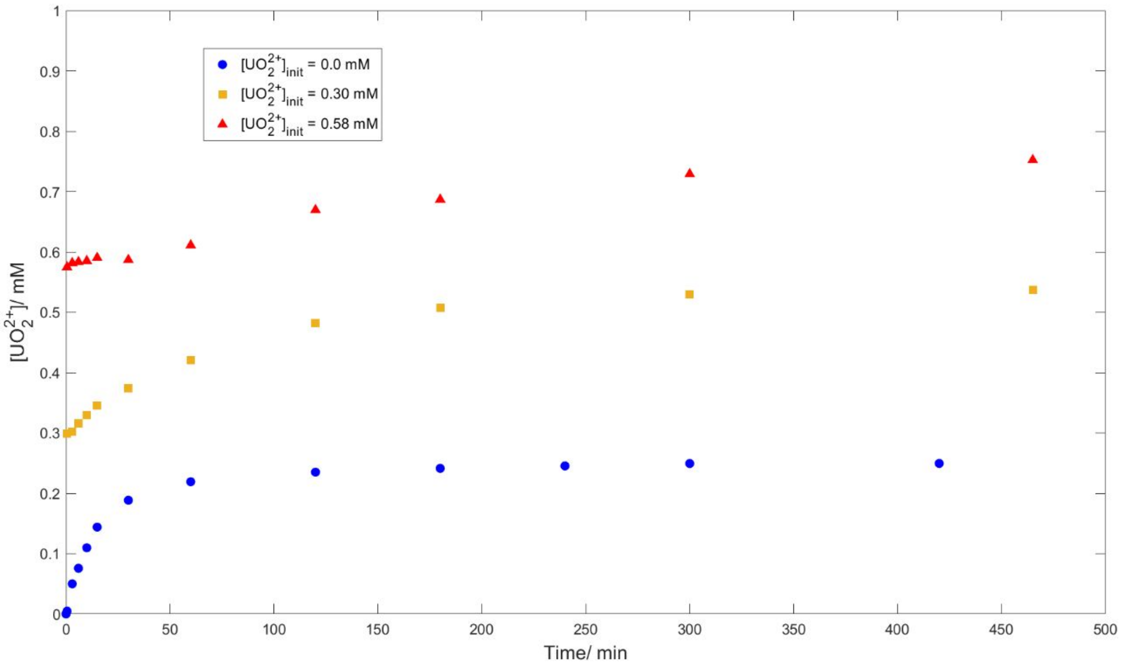

Figure S5. Measured uranyl concentrations vs time for the data presented in Figure 2.

Scale up of Figure 6 (b)

A scale-up showing the comparison between experimental rates (solid lines) and calculated rates (dashed lines) for the two systems where an initial amount of $[UO_2^{2+}]$ was added.

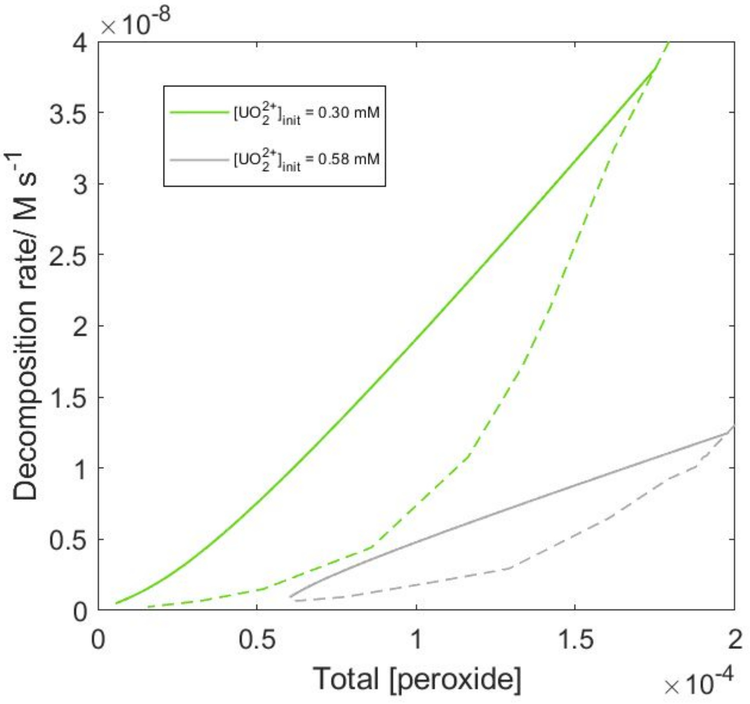

Figure S6. Scaleup of the two systems with added initial [UO_2^{2+}], presented in Figure 6 (b).

Titration data for the Arsenazo-III method

Calibration data for the measurement of Arsenazo-III-U(VI) at 653 nm is presented in Figure S7.

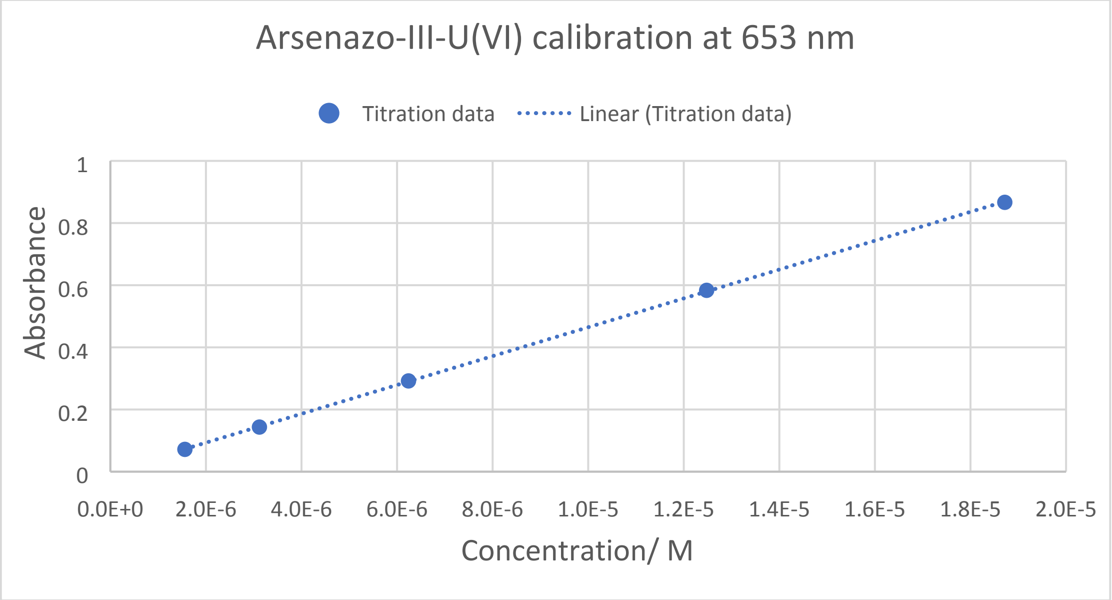

Figure S7. Calibration data from aqueous uranyl-nitrate titration, measured at 653 nm.

Data from thermodynamic calculations

The speciation data from thermodynamic calculations in SPANA,[2] for 100 mg UO_2 powder in 100 ml 10 mM bicarbonate solution and varied initial [H_2O_2] have been listed in Tables S1 to S4. The calculations are based on the measured concentrations of UO_2^{2+} and H_2O_2, ionic strength correction using the SIT model and stability constants published by Zanonato *et al.*[3] The data includes the three dominant species listed as A, B and C, corresponding to free H_2O_2, $UO_2(O_2)(CO_3)_4^{6-}$ and $UO_2(O_2)(CO_3)_2^{4-}$ respectively. We have included the data for several pH as no pH had been reported in the original study (Barreiro Fidalgo *et al.*)[1].

Table S1. Data from speciation calculations for 0.2 mM initial [H_2O_2] for pH 7, 8, 9, 10 and 11 using ionic strength correction based on the SIT model.

Initial H_2O_2 concentration	pH*	Time/ s	Concentrations measured with UV-vis/ *10^{-4} M		Fractions based on thermodynamic equilibrium			Equilibrium concentrations of peroxide species/ *10^{-3} M		
			[Peroxide]	[UO_2^{2+}]	A	B	C	A	B	C
0.2 mM	7	0	1.98	0	1	0	0	1.98	0.00	0.00
		30	1.76	0.0274	1	0	0	1.76	0.00	0.00
		600	0.941	0.603	0.880	0.074	0	0.83	0.07	0.00
		1200	0.590	1.11	0.691	0.218	0	0.41	0.13	0.00
		1800	0.413	1.46	0.534	0.346	0	0.22	0.14	0.00
		2400	0.300	1.63	0.446	0.420	0	0.13	0.13	0.00
		3600	0.200	1.77	0.373	0.483	0	0.07	0.10	0.00
		5130	0.117	1.91	0.312	0.537	0	0.04	0.06	0.00
	8	0	1.98	0	1	0	0	1.98	0.00	0.00
		30	1.76	0.0274	1	0	0	1.76	0.00	0.00
		600	0.941	0.603	0.870	0.085	0	0.82	0.08	0.00
		1200	0.590	1.11	0.675	0.253	0	0.40	0.15	0.00
		1800	0.413	1.46	0.5200	0.404	0	0.21	0.17	0.00
		2400	0.300	1.63	0.433	0.492	0	0.13	0.15	0.00
		3600	0.200	1.77	0.359	0.561	0	0.07	0.11	0.00
		5130	0.117	1.91	0.302	0.625	0	0.04	0.07	0.00
	9	0	1.98	0	1	0	0	1.98	0.00	0.00
		30	1.76	0.0274	1	0	0	1.76	0.00	0.00
		600	0.941	0.603	0.796	0.076	0.094	0.75	0.07	0.09
		1200	0.590	1.11	0.563	0.250	0.248	0.33	0.15	0.15
		1800	0.413	1.46	0.400	0.400	0.151	0.17	0.17	0.06
		2400	0.300	1.63	0.318	0.483	0.144	0.10	0.14	0.04
		3600	0.200	1.77	0.259	0.552	0.139	0.05	0.11	0.03
		5130	0.117	1.91	0.208	0.609	0.133	0.02	0.07	0.02
	10	0	1.98	0	1	0	0	1.98	0.00	0.00
		30	1.76	0.0274	1	0	0	1.76	0.00	0.00
		600	0.941	0.603	0.497	0.031	0.438	0.47	0.03	0.41
		1200	0.590	1.11	0.147	0.208	0.609	0.09	0.12	0.36
		1800	0.413	1.46	0.07	0.356	0.541	0.03	0.15	0.22
		2400	0.300	1.63	0.047	0.429	0.490	0.01	0.13	0.15
		3600	0.200	1.77	0.036	0.483	0.453	0.01	0.10	0.09
		5130	0.117	1.91	0.028	0.527	0.420	0.00	0.06	0.05
	11	0	1.98	0	1	0	0	1.98	0.00	0.00
		30	1.76	0.0274	1	0	0	1.76	0.00	0.00
		600	0.941	0.603	0.369	0	0.612	0.35	0.00	0.58
		1200	0.590	1.11	0	0.173	0.785	0.00	0.10	0.46
		1800	0.413	1.46	0	0.319	0.646	0.00	0.13	0.27
		2400	0.300	1.63	0	0.380	0.578	0.00	0.11	0.17
		3600	0.200	1.77	0	0.427	0.532	0.00	0.09	0.11
		5130	0.117	1.91	0	0.466	0.492	0.00	0.05	0.06

Table S2. Data from speciation calculations for 0.5 mM initial [H_2O_2] for pH 7, 8, 9, 10 and 11 using ionic strength correction based on the SIT model.

Initial H_2O_2 concentration	pH*	Time/ s	Concentrations measured with UV-vis/ *10^{-4} M		Fractions based on thermodynamic equilibrium			Equilibrium concentrations of peroxide species/ *10^{-4} M		
			[Peroxide]	[UO_2^{2+}]	A	B	C	A	B	C
0.5 mM	7	0	5.0	0	1	0	0	5.00	0.00	0.00
		30	4.545	0.140	1	0	0	4.55	0.00	0.00
		600	2.905	0.809	0.905	0.117	0	2.63	0.34	0.00
		1200	1.996	1.45	0.768	0.158	0	1.53	0.32	0.00
		1800	1.470	2.11	0.583	0.306	0	0.86	0.45	0.00
		2400	1.162	2.58	0.433	0.435	0	0.50	0.51	0.00
		3000	0.981	2.88	0.335	0.520	0	0.33	0.51	0.00
		3600	0.842	3.11	0.265	0.583	0	0.22	0.49	0.00
		4500	0.700	3.23	0.219	0.626	0	0.15	0.44	0.00
		6720	0.502	3.57	0.148	0.689	0	0.07	0.35	0.00
	8	0	5.0	0	1	0	0	5.00	0.00	0.00
		30	4.545	0.140	1	0	0	4.55	0.00	0.00
		600	2.905	0.809	0.899	0.136	0	2.61	0.40	0.00
		1200	1.996	1.45	0.759	0.181	0	1.51	0.36	0.00
		1800	1.470	2.11	0.572	0.353	0	0.84	0.52	0.00
		2400	1.162	2.58	0.417	0.507	0	0.48	0.59	0.00
		3000	0.981	2.88	0.319	0.606	0	0.31	0.59	0.00
		3600	0.842	3.11	0.253	0.677	0	0.21	0.57	0.00
		4500	0.700	3.23	0.208	0.726	0	0.15	0.51	0.00
		6720	0.502	3.57	0.142	0.800	0	0.07	0.40	0.00
	9	0	5.0	0	1	0	0	5.00	0.00	0.00
		30	4.545	0.140	0.979	0	0.002	4.45	0.00	0.01
		600	2.905	0.809	0.853	0.107	0.107	2.48	0.31	0.31
		1200	1.996	1.45	0.680	0.156	0.122	1.36	0.31	0.24
		1800	1.470	2.11	0.475	0.329	0.145	0.70	0.48	0.21
		2400	1.162	2.58	0.322	0.481	0.147	0.37	0.56	0.17
		3000	0.981	2.88	0.236	0.580	0.138	0.23	0.57	0.14
		3600	0.842	3.11	0.181	0.648	0.127	0.15	0.55	0.11
		4500	0.700	3.23	0.147	0.694	0.117	0.10	0.49	0.08
		6720	0.502	3.57	0.099	0.768	0.100	0.05	0.39	0.05
	10	0	5.0	0	1	0	0	5.00	0.00	0.00
		30	4.545	0.140	0.979	0	0.022	4.45	0.00	0.10
		600	2.905	0.809	0.544	0	0.398	1.58	0.00	1.16
		1200	1.996	1.45	0.398	0.056	0.512	0.79	0.11	1.02
		1800	1.470	2.11	0.134	0.222	0.603	0.20	0.33	0.89
		2400	1.162	2.58	0.057	0.395	0.518	0.07	0.46	0.60
		3000	0.981	2.88	0.034	0.487	0.449	0.03	0.48	0.44
		3600	0.842	3.11	0.023	0.551	0.401	0.02	0.46	0.34
		4500	0.700	3.23	0.02	0.592	0.367	0.01	0.41	0.26
		6720	0.502	3.57	0.016	0.657	0.313	0.01	0.33	0.16
	11	0	5.0	0	1	0	0	5.00	0.00	0.00
		30	4.545	0.140	0.978	0	0.027	4.45	0.00	0.12
		600	2.905	0.809	0.503	0	0.481	1.46	0.00	1.40
		1200	1.996	1.45	0.279	0	0.694	0.56	0.00	1.39
		1800	1.470	2.11	0.002	0.168	0.793	0.00	0.25	1.17
		2400	1.162	2.58	0	0.343	0.618	0.00	0.40	0.72
		3000	0.981	2.88	0	0.430	0.526	0.00	0.42	0.52
		3600	0.842	3.11	0	0.487	0.467	0.00	0.41	0.39
		4500	0.700	3.23	0	0.527	0.432	0.00	0.37	0.30
		6720	0.502	3.57	0	0.585	0.369	0.00	0.29	0.19

Table S3. Data from speciation calculations for 1.0 mM initial [H_2O_2] for pH 7, 8, 9, 10 and 11 using ionic strength correction based on the SIT model.

Initial H_2O_2 concentration	pH*	Time/ s	Concentrations measured with UV-vis/ *10^{-4} M		Fractions based on thermodynamic equilibrium			Equilibrium concentrations of peroxide species/ *10^{-3} M		
			[Peroxide]	[UO_2^{2+}]	A	B	C	A	B	C
1.0 mM	7	0	10.0	0				10.00	0.00	0.00
		30	9.32	0.0913	1	0	0	9.32	0.00	0.00
		600	6.72	0.902	1	0	0	6.72	0.00	0.00
		1200	5.00	1.72	0.853	0.093	0	4.27	0.47	0.00
		1800	3.94	2.57	0.734	0.185	0	2.89	0.73	0.00
		2400	3.08	3.29	0.588	0.302	0	1.81	0.93	0.00
		3000	2.65	3.38	0.534	0.353	0	1.42	0.94	0.00
		3600	2.37	4.41	0.358	0.511	0	0.85	1.21	0.00
		4500	2.09	4.79	0.265	0.591	0	0.55	1.24	0.00
		6720	1.77	5.18	0.180	0.672	0	0.32	1.19	0.00
		12600	1.29	5.63	0.094	0.753	0	0.12	0.97	0.00
	8	0	10.0	0				0.00	0.00	0.00
		30	9.32	0.0913	1	0	0	9.32	0.00	0.00
		600	6.72	0.902	1	0	0	6.72	0.00	0.00
		1200	5.00	1.72	0.845	0.105	0	4.23	0.53	0.00
		1800	3.94	2.57	0.723	0.210	0	2.85	0.83	0.00
		2400	3.08	3.29	0.578	0.349	0	1.78	1.07	0.00
		3000	2.65	3.38	0.518	0.404	0	1.37	1.07	0.00
		3600	2.37	4.41	0.343	0.583	0	0.81	1.38	0.00
		4500	2.09	4.79	0.253	0.677	0	0.53	1.41	0.00
		6720	1.77	5.18	0.168	0.773	0	0.30	1.37	0.00
		12600	1.29	5.63	0.09	0.865	0	0.12	1.12	0.00
	9	0	10.0	0	1	0	0	10.00	0.00	0.00
		30	9.32	0.0913	1	0	0	9.32	0.00	0.00
		600	6.72	0.902	0.857	0.045	0.076	5.76	0.30	0.51
		1200	5.00	1.72	0.797	0.077	0.093	3.99	0.39	0.47
		1800	3.94	2.57	0.655	0.178	0.125	2.58	0.70	0.49
		2400	3.08	3.29	0.497	0.309	0.145	1.53	0.95	0.45
		3000	2.65	3.38	0.435	0.367	0.150	1.15	0.97	0.40
		3600	2.37	4.41	0.264	0.548	0.141	0.63	1.30	0.33
		4500	2.09	4.79	0.187	0.642	0.128	0.39	1.34	0.27
		6720	1.77	5.18	0.119	0.740	0.107	0.21	1.31	0.19
		12600	1.29	5.63	0.06	0.831	0.082	0.08	1.07	0.11
	10	0	10.0	0	1	0	0	10.00	0.00	0.00
		30	9.32	0.0913	1	0	0	9.32	0.00	0.00
		600	6.72	0.902	0.848	0	0.164	5.70	0.00	1.10
		1200	5.00	1.72	0.666	0	0.296	3.33	0.00	1.48
		1800	3.94	2.57	0.424	0.048	0.492	1.67	0.19	1.94
		2400	3.08	3.29	0.195	0.154	0.606	0.60	0.47	1.87
		3000	2.65	3.38	0.134	0.227	0.600	0.36	0.60	1.59
		3600	2.37	4.41	0.048	0.433	0.492	0.11	1.03	1.17
		4500	2.09	4.79	0.027	0.537	0.410	0.06	1.12	0.86
		6720	1.77	5.18	0.518	0.629	0.336	0.92	1.11	0.59
		12600	1.29	5.63	0.008	0.722	0.256	0.01	0.93	0.33
								0.00	0.00	0.00
	11	0	10.0	0	1	0	0	10.00	0.00	0.00
		30	9.32	0.0913	1	0	0	9.32	0.00	0.00
		600	6.72	0.902	1	0	0	6.72	0.00	0.00
		1200	5.00	1.72	0.661		0.332	3.31	0.00	1.66
		1800	3.94	2.57	0.352	0	0.625	1.39	0.00	2.46
		2400	3.08	3.29	0.016	0.053	0.893	0.05	0.16	2.75
		3000	2.65	3.38	0	0.151	0.806	0.00	0.40	2.14
		3600	2.37	4.41	0	0.376	0.581	0.00	0.89	1.38
		4500	2.09	4.79	0	0.477	0.479	0.00	1.00	1.00

		6720	1.77	5.18	0	0.561	0.392	0.00	0.99	0.69
		12600	1.29	5.63	0	0.646	0.302	0.00	0.83	0.39

Table S4. Data from speciation calculations for 2.0 mM initial [H_2O_2] for pH 7, 8, 9, 10 and 11 using ionic strength correction based on the SIT model.

Initial H_2O_2 concentration	pH*	Time/ s	Concentrations measured with UV-vis/ *10^{-4} M		Fractions based on thermodynamic equilibrium			Equilibrium concentrations of peroxide species/ *10^{-3} M		
			[Peroxide]	[UO_2^{2+}]	A	B	C	A	B	C
2.0 mM	7	0	19.4	0.107	1	0	0	19.40	0.00	0.00
		660	14.9	1.02	1	0	0	14.90	0.00	0.00
		1260	12.0	1.97	0.916	0.045	0	10.99	0.54	0.00
		1800	9.56	2.64	0.862	0.08	0	8.24	0.76	0.00
		2400	7.78	3.41	0.737	0.181	0	5.73	1.41	0.00
		3300	5.86	4.54	0.648	0.25	0	3.80	1.47	0.00
		4560	4.31	5.80	0.438	0.438	0	1.89	1.89	0.00
		6720	3.41	7.00	0.23	0.634	0.093	0.78	2.16	0.32
	8	0	19.4	0.107	1	0	0	19.40	0.00	0.00
		660	14.9	1.02	1	0	0	14.90	0.00	0.00
		1260	12.0	1.97	0.910	0.051	0	10.92	0.61	0.00
		1800	9.56	2.64	0.853	0.9	0	8.15	8.60	0.00
		2400	7.78	3.41	0.725	0.201	0	5.64	1.56	0.00
		3300	5.86	4.54	0.635	0.278	0	3.72	1.63	0.00
		4560	4.31	5.80	0.421	0.495	0	1.81	2.13	0.00
		6720	3.41	7.00	0.218	0.719	0	0.74	2.45	0.00
	9	0	19.4	0.107	1	0	0	19.40	0.00	0.00
		660	14.9	1.02	0.947	0	0.036	14.11	0.00	0.54
		1260	12.0	1.97	0.882	0.031	0.064	10.58	0.37	0.77
		1800	9.56	2.64	0.816	0.062	0.087	7.80	0.59	0.83
		2400	7.78	3.41	0.665	0.165	0.122	5.17	1.28	0.95
		3300	5.86	4.54	0.566	0.236	0.133	3.32	1.38	0.78
		4560	4.31	5.80	0.346	0.452	0.144	1.49	1.95	0.62
		6720	3.41	7.00	0.158	0.682	0.119	0.54	2.33	0.41
	10	0	19.4	0.107	1	0	0	19.40	0.00	0.00
		660	14.9	1.02	0.919	0	0.065	13.69	0.00	0.97
		1260	12.0	1.97	0.828	0	0.150	9.94	0.00	1.80
		1800	9.56	2.64	0.723	0	0.215	6.91	0.00	2.06
		2400	7.78	3.41	0.467	0.036	0.452	3.63	0.28	3.52
		3300	5.86	4.54	0.324	0.074	0.534	1.90	0.43	3.13
		4560	4.31	5.80	0.093	0.296	0.558	0.40	1.28	2.40
		6720	3.41	7.00	0.023	0.569	0.384	0.08	1.94	1.31
	11	0	19.4	0.107	1	0	0	19.40	0.00	0.00
		660	14.9	1.02	0.934	0	0.068	13.92	0.00	1.01
		1260	12.0	1.97	0.835	0	0.159	10.02	0.00	1.91
		1800	9.56	2.64	0.718	0	0.265	6.86	0.00	2.53
		2400	7.78	3.41	0.366	0	0.551	2.85	0.00	4.29
		3300	5.86	4.54	0.196	0	0.712	1.15	0.00	4.17
		4560	4.31	5.80	0	0.579	0.725	0.00	2.50	3.12
		6720	3.41	7.00	0	0.504	0.447	0.00	1.72	1.52

Pseudo-first order rate constants for 100 mg $UO_{2.3}$ powder in 100 cm^3 (10 mM bicarbonate) with initial [H_2O_2] of 0.2, 0.5, 1.0 and 2.0 mM have been listed in Table S5. The constants are calculated as the initial peroxide consumption rates divided by the initial peroxide concentrations.

**Table S5. Pseudo-first order rate constants for systems with varied initial [H_2O_2]**	
Initial [H_2O_2]	Pseudo-first order rate constant (k') [s^{-1}]
0.2 mM	$1.284 * 10^{-3}$
0.5 mM	$8.906 * 10^{-4}$
1 mM	$6.381 * 10^{-4}$
2 mM	$3.952 * 10^{-4}$

REFERENCES

(1) A. Barreiro Fidalgo, Y. Kumagai, and M. Jonsson, "The role of surface-bound hydroxyl radicals in the reaction between H_2O_2 and UO_2," *J. Coord. Chem.,* vol. 71, no. 11-13, pp. 1799-1807, 2018, doi: 10.1080/00958972.2018.1466287.

(2) I. Puigdomènech, E. Colàs, M. Grivé, I. Campos, and D. García, "A tool to draw chemical equilibrium diagrams using SIT: Applications to geochemical systems and radionuclide solubility," *MRS Online Proc. Libr.,* vol. 1665, pp. 111-116, 2014, doi: 10.1557/opl.2014.635.

(3) P. L. Zanonato, P. Di Bernardo, Z. Szabó, and I. Grenthe, "Chemical equilibria in the uranyl(vi)–peroxide–carbonate system; identification of precursors for the formation of poly-peroxometallates," *Dalton Trans.,* vol. 41, no. 38, p. 11635-11641, 2012, doi: 10.1039/c2dt31282d.
